# Motives for early retirement of self-employed GPs in the Netherlands: a comparison of two time periods

**DOI:** 10.1186/1472-6963-12-467

**Published:** 2012-12-18

**Authors:** Malou Van Greuningen, Phil JM Heiligers, Lud FJ Van der Velden

**Affiliations:** 1 NIVEL, Netherlands Institute for Health Services Research, P.O. Box 1568, 3500, BN, Utrecht, the Netherlands; 2 Utrecht University, Faculty of Social Sciences, P.O. Box 80.140, 3508, TC, Utrecht, the Netherlands

**Keywords:** Retirement, Health workforce, Health workforce planning, Workload reduction, Policy

## Abstract

**Background:**

The high cost of training and the relatively long period of training for physicians make it beneficial to stimulate physicians to retire later. Therefore, a better understanding of the link between the factors influencing the decision to retire and actual turnover would benefit policies designed to encourage later retirement. This study focuses on actual GP turnover and the determining factors for this in the Netherlands. The period 2003–2007 saw fewer GPs retiring from general practice than the period 1998–2002. In addition, GPs’ retirement age was higher in 2003–2007. For these two periods, we analysed work perception, objective workload and reasons for leaving, and related these with the probability that GPs would leave general practice at an early age.

**Methods:**

In 2003, a first retrospective survey was sent to 520 self-employed GPs who had retired between 1998 and 2002. In 2008, the same survey was sent to 405 GPs who had retired between 2003 and 2007. The response rates were 60% and 54%, respectively. Analyses were done to compare work perception, objective workload, external factors and personal reasons for retiring.

**Results:**

For both male and female GPs, work perception was different in the periods under scrutiny: both groups reported greater job satisfaction and a lower degree of emotional exhaustion in the later period, although there was no notable difference in subjective workload. The objective workload was lower in the second period. Moreover, most external factors and personal reasons that may contribute to the decision to retire were reported as less important in the second period. There was a stronger decrease in the probability that female GPs leave general practice within one year than for male GPs. This underscores the gender differences and the need for disaggregated data collection.

**Conclusions:**

The results of this study suggest that the decrease in the probability of GPs leaving general practice within one year and the increasing retirement age are caused by a decrease in the objective workload, a change in GPs’ work perception, external factors and personal reasons. Based on the results of this study, we consider workload reduction policies are the most useful instruments to control retention and retirement.

## Introduction

Many countries face challenges in matching the supply and demand for health professionals. With gaps already appearing between the supply of and the demand for health professionals in some countries, it is important to understand what future trends might affect supply and demand over the next 20 years [1]. Since the beginning of this century, many policy reports mention shortages on health labour markets as one of the most urgent problems [2-5]. The challenge of matching supply with demand for physicians and other health professionals include making the ‘right’ decisions on the inflow in training, on the retention and retirement of the existing stock of physicians, and on migration policies for physicians. Countries have a variety of additional policy instruments at their disposal to influence the supply of physicians including education and training policies, migration policies and policies affecting retention and retirement [1].

### Health workforce planning

It is commonly acknowledged that workforce planning is an important policy instrument used to control shortages and oversupply within the health care labour market [6-11]. In the Netherlands, a simulation model is used to match the supply and demand for health professionals. This model determines the required number of health professionals in training to meet the future demand for care [12]. There are several factors that influence the future supply of health professionals : the present and future number of health professionals in stock (male/female), the amount of full-time equivalent (FTE) they work (present and future), the inflow from training, the inflow from abroad and the outflow of health professionals. The retirement age of physicians and other health professionals provides important information for workforce planning; after all, the projections of the future outflow of health professionals are based on the retirement age of health professionals in the past.

### Ageing of the health workforce

The ageing of the physician population is likely to have a profound effect on the outflow of physicians and thus the future supply of physicians in many countries. The generation of doctors who were born during the ‘baby boom’ following World War II, will be coming up to retirement and leave the profession during the next decade or two [1]. Due to the high training cost and the relatively long training period for physicians, it is important to stimulate physicians to retire at an older age to maintain a sufficient number of physicians. In addition, a greater understanding of the link between the factors influencing the decision to retire and actual turnover would benefit policies designed to encourage later retirement.

### Early retirement among Dutch general practitioners (GPs)

Dutch GPs are increasingly choosing to work part-time and are leaving their profession at a relatively early age [13,14]. Early retirement among GPs reflects a wider societal trend towards early retirement seen in the past; however, this societal trend is now changing as the number of people who are willing to work until 65 (statutory retirement age) is increasing in the Netherlands [15]. This raises the question whether Dutch GPs are also following this trend.

GPs are the focus of our study as they play an important role within the Dutch health care system [16] and represent one of the largest professional groups within the health workforce. They provide primary health care twenty-four hours a day, seven days a week. In addition, they are the gatekeepers of the Dutch health care system. In other words, it is important to have sufficient GPs in the Netherlands to meet the demand for care [17].

To investigate whether Dutch GPs’ retirement age has become higher in the preceding years, firstly, this article explores the difference between the periods 1998–2002 and 2003–2007 regarding the probability of leaving general practice within one year (explained more extensively in the background section) and GPs’ retirement age. Secondly, we investigate to what extent work perception (job satisfaction, emotional exhaustion, *subjective* workload), *objective* workload, external factors and personal reasons changed between these two periods and if these changes influenced the decision to leave general practice in 1998–2002 and 2003–2007. The reasons that can contribute to the decision to retire are described more extensively in the methods section of this article.

In this study, the focus lies on exploring the differences between 1998–2002 and 2003–2007. The aim of this study is not to test the relationships or interactions between the determining factors and the probability to leave or the retirement age, because these relationships have been examined extensively in earlier studies which will be presented in the background section and the methods section of this article.

The probability of leaving general practice, the retirement age and factors influencing the decision to retire are different for male and female GPs, and therefore the differences between male and female GPs are also looked into [18-22]. The article focuses on GPs who have already left general practice. The following research questions are investigated:

1. Did the work perception (job satisfaction, emotional exhaustion, *subjective* workload) of GPs change between 1998–2002 and 2003-2007? Did male and female GPs report differently on work perception?

2. Did the *objective* workload of GPs change between 1998–2002 and 2003-2007? Did male and female GPs report differently on *objective* workload?

3. Which external factors and personal reasons do GPs report as being the most important contributors to their decision to leave general practice in both periods? Did the extent to which external factors and personal reasons contributed to the decision to leave the profession change between 1998–2002 and 2003-2007? Did male GPs and female GPs report differently on these reasons in these periods?

4) Is there a relation between, on the one hand, the probability of retirement from general practice within one year and GPs’ retirement age and, on the other hand, the reported reasons for leaving and work perception and workload?

In the following sections, the (inter)national context of this study is discussed, as well as the design of the study. Subsequently, the questionnaire is discussed, which was based on earlier research on work perception, objective workload, external factors and personal reasons that may influence the decision to leave general practice early. Then, the results of the analyses are described. Finally, the results are summarised and conclusions are drawn.

## Background

### International context

Demographic changes in Europe, especially the on-going process of population ageing, increase demands for health services while simultaneously shrinking the pool of workers available to offer these services. The workforce in OECD countries is ageing as the ‘baby boom’ generation of health workers begins to reach retirement age [4,23]. As these staff approach retirement age they need to be replaced by sufficient younger health workers. Between 1995 and 2000, the number of physicians under the age of 45 across Europe dropped by 20%, whilst the number ages over 45 went up by over 50%, which means that the ‘baby boom’ generation accounts for a substantial share of the health workforce [24]. Several studies have shown that the proportion of physicians working beyond the age of 60 years has fallen in most European countries over the past decade [25-27]. Until recently, few OECD countries had implemented or planned specific policies to address this issue [28]. But regarding the health workforce, there have been some attempts to reverse the trend towards early retirement and retain older workers within the workforce. In the United Kingdom for example, a flexible-retirement initiative, launched in 2000, enabled staff nearing retirement to move into part-time work while preserving pension entitlements [1]. And in France, doctors who reach the statutory pensionable age can combine a pension and earnings up to an income limit. Also, elderly doctors can be exempted from night and weekend shifts [29].

Also in the Netherlands, in the near future, the demand for care is expected to increase due to the aging population and an increasing number of chronically ill patients [17]. In combination with the shift from hospital care to primary care, this will put pressure on primary health care professionals, for example the general practitioner (GP), who is the gatekeeper of the Dutch health care system [30,31]. These trends in health care demand are accompanied by developments in health care supply. There are likely to be reductions in the availability of physicians in most countries unless steps are taken to increase recruitment. That is mainly because of changes in lifetime hours worked, increasing female participation in the workforce, increasing specialisation, physician workforce ageing and a growing number of retirements [1,4,17].

### Dutch health workforce planning system

The health care system in the Netherlands is rooted in the “Bismarckian” social insurance tradition. When the 2006 Health Insurance Act was introduced, the distinction between mandatory sickness fund insurance and voluntary private insurance, which had existed in the Netherlands since the Second World War, was changed to a system with a basic insurance for all citizens [16,17].

In the Netherlands, the Advisory Committee on Medical Manpower Planning (*Capaciteitsorgaan*), established in 1999, is an independent advisory committee which focuses on determining the medical training capacity required to meet the demand for care. The Advisory Committee is informed by a workforce forecasting model for physicians (developed by NIVEL). This model is based on realizing an equilibrium in projection (i.e. for 2020, 2025) based on assumptions, heuristics and statistics about the supply and demand side of the health care labour market. The output of the planning model is a calculation of the required yearly inflow in medical training within the next five to fifteen years. After these calculations, the results of the model are discussed within specialized platforms of the Advisory Committee on Medical Manpower Planning, which consists of representatives of professionals, health insurers and the medical training institutions [32]. When the model simulations, including the different scenarios, have been carried out, the draft inflow recommendations are discussed by the plenary platform of the Advisory Committee. This platform determines the advice to give to the Ministry regarding the yearly inflow in training for health professionals. This advice is subsequently discussed with the Ministry of Health, Welfare and Sport. After the Ministry and national government have decided on the total budget for the training of all (academic) health professionals, this budget steers the advice medical faculties, schools and universities receive on their annual student enrolment number [12,16].

Among the key requirements for human resource planning in the health sector are accurate and comprehensive information systems on the actual number of health care workers and their distribution in the health care system [23]. Data on a variety of topics is necessary for adequate workforce planning for health professionals, as there are many factors that influence the size of the workforces of GPs and other health professionals. NIVEL administers a large database with information about Dutch GPs, for example, their gender, age, retirement age, etcetera. This information is used to inform the planning of the Dutch GP workforce. In addition to this database, in-depth studies regarding different elements of the model are conducted to keep assumptions about the models’ elements up-to-date.

### Retirement and probability to leave general practice in 1998–2002 and 2003–2007

According to Van der Velden & Batenburg [22], between 1998 and 2002, 1,135 self-employed GPs left general practice in the Netherlands, 206 (18.1%) of whom were female and 929 (81.9%) male. Between 2003 and 2007, 998 self-employed GPs left general practice, 177 (17.7%) of whom were female and 821 (82.3%) male [22]. Based on this information, the probability of retiring within one year was calculated. The probability of retiring is equal to the percentage of self-employed GPs that leave general practice (per year, and subdivided by age group and gender). There is a difference in the probability of leaving general practice in the Netherlands within one year for the periods 1998–2002 and 2003–2007 [22]. In the period 1998–2002, this probability was 1.7% for GPs younger than 55. This means that of every 100 active GPs (younger than 55) at the beginning of a certain year, an average of 1.7 GPs had left general practice at the end of that same year. For GPs between 55 and 59 years old, the probability of leaving within one year was 7.6%, and between 60 and 64 years old it was 35.1%. The statutory retirement age in the Netherlands is 65. In the second period, 2003–2007, the probability of leaving was lower for all age groups: 0.9%, 5.2% and 21.3%, respectively. For female GPs, the difference between both periods was larger than for male GPs. In Additional file 1, the probability of leaving is presented. Furthermore, according to the same study [22], the retirement age of both male and female GPs was lower in the second period (M: 58.8, F: 49.1) than in the first period (M: 56.5, F: 47.0). In summary, fewer GPs retired from general practice in the period 2003–2007 than in the period 1998–2002, and the retirement age of these GPs was higher in 2003–2007 than in the period 1998–2002. This information reflects the entire GP population in the Netherlands in those periods. Various reasons influence the decision to leave general practice. A changing probability to leave general practice could be based on a changing influence of these explanatory factors on the decision to retire.

Several studies have investigated the relation between explanatory factors and both the physicians’ intentions to leave practice and their actual leaving [21,33-35]. In these studies we found several factors that may help explain early retirement among GPs, for example external factors (such as government policy), personal characteristics, job-related perception and objective workload. Job satisfaction and workload are measured in many different ways [36]. In this study, job satisfaction and subjective workload are both a work perception-measure, while objective workload is a numeric measure: number of working hours. These measures are further described in the methods section.

Only a small number of studies have investigated the effect of job satisfaction or other possible factors on actual physician turnover, rather than examining their effect on the intention to leave [37-39]. For example, Rittenhouse et al. [40] found that the strongest predictor of both intention to leave practice and actual leaving was, not surprisingly, advancing age. However, the intentions to leave and actual leaving may also be influenced by factors unrelated to the occupation, such as health, the need to care for a dependent relative or the desire to take a career break to raise children [34]. The factors that may help explain early retirement among GPs are further described in the methods section of this article.

### GP workload reduction policies in the Netherlands

Besides the use of GP workforce planning policies to realize an equilibrium between supply and demand, which is a long-term solution for avoiding shortages of GPs, other, short-term policies can be implemented to prevent the early retirement of GPs. Several workload reduction measures were introduced in the Netherlands at the start of the 21st century. These measures were implemented to reduce high workload among GPs. This was a response to the dissatisfaction among GPs regarding their workload, which led to a series of nationwide campaigns and even to a one-day strike [41,42]. The introduction of these measures could also be a possible explanation for differences found between the two time periods, regarding GPs’ retirement age and changes in the influence of explanatory factors. Both workload reduction measures are discussed below.

First, there was the nationwide introduction of central GP services for evening, night and weekend service. Traditionally, the GP used to work alone, but since the 1970s group practices have become popular. Until the late 1990s, out-of-hours care was organized by small groups of GPs, where each GP had night or weekend shifts on a regular basis. Since the early 2000s, out-of-hours care has become organized in a national network of so-called GP posts. A GP post is a centrally located office with a GP present after hours [43]. This change from small- to large-scale service structures took place in the late nineties; in 2002, 80- 90% of all GPs were associated with a large GP service center [44]. In earlier research, 80% of all GPs mentioned the evening, night and weekend shifts as a highly stressful job demand [45,46]. Furthermore, these out-of-hours shifts in small-scale settings were mentioned as the most important reason to retire before the age of 60 [47]. After the introduction of large-scale central GP services, the number of shifts per GP and the experienced workload reduced significantly [44,48]. The reduction of out-of-hours shifts is thus associated with both a decrease in objective and subjective workload.

The second development was the introduction of general practice nurses in GP practices in 1999. This new type of function was one of the measurements taken in a broader plan of job reallocation in health care. The underlying reasons were an increasing demand for care by an aging population, combined with a decrease in the number of health professionals and a growing number of part-time workers [49]. Another reason for the introduction of task reallocation was the growing pressure to improve quality of care. A possible reduction in GP workload was expected if GPs could focus on their core tasks and the provision of care was shared with other health professionals.

The introduction of practice nurses is part of the trend that is commonly called “substitution”. This can be defined as the (partial) vertical transfer of tasks from doctors to nurses, and horizontal task re-allocation between groups of health care workers. Substitution is mainly driven by efficiency, but can also be seen as inevitable in order to cope with the increasing physician workload [16,17].

In the following sections the design of the present study and its results will be discussed.

## Methods

### Design and Subjects

To collect data in this study, a retrospective postal survey was sent to previously self-employed general practitioners in the Netherlands who had retired from their work as GPs before the age of 65, which is the statutory pensionable age in the Netherlands. This survey was sent to a random sample (520) drawn from the population of GPs that had retired between January 1998 and December 2002 (a total of 1,135 former GPs). Data were collected in the Netherlands in the winter of 2003/2004. The response rate was 60%.

The same survey was sent in the winter of 2008/2009 to a second group of previously self-employed GPs in the Netherlands. The GPs included in this random sample (405) were drawn from the population of GPs who had retired between January 2003 and December 2007 (a total of 998 former GPs). The response rate of this survey was 54%. Potential participants for both periods were identified using data files from the GP database of NIVEL.

In the Netherlands, there are different types of GPs: GPs who have their own practice are self-employed, and GPs who are employed by another GP and who do not have their own practice. The latter are called salaried GPs [16]. The samples only comprised self-employed GPs and not salaried GPs, as the effects of early retirement are likely to be different for these two types of GPs. For salaried GPs, it is probably easier to leave general practice because they do not have their own practice and another GP can easily take over their job. Analysis of the differences in personal characteristics between respondents and non-respondents showed that on average the respondents were somewhat older than the entire population of GPs leaving general practice (Table 1).

**Table 1:** Population and respondents: mean retirement age and gender

	**Male**		**Female**	
**Period**	**Population**	**Respondents**	**Population**	**Respondents**
	**(n = 1,135)**	**(n = 282)**	**(n = 998)**	**(n = 191)**
1998-2002	56.5	58.0	47.0	53.9
2003-2007	58.8	61.0	49.1	53.5

### Measures

The survey was constructed using input from different studies (referred to in the following paragraphs) and included the most relevant factors concerning early retirement and job satisfaction. The scales used in this study are also discussed in the next few paragraphs. GPs that had left general practice before they reached the retirement age of 65 were asked retrospectively to rate their work perception and their experience with external factors and reasons for leaving general practice, as well as to report on their hours worked. It is possible that their answers have been influenced by the time elapsed. However, we expect this bias to be minimal, because it has been shown that reports on jobs that respondents had five years ago do not exhibit greater unreliability than reports on jobs they had a few months ago [50,51]. Respondents received the survey a maximum of 5 years after they retired.

#### Work perception

Several aspects of work perception have been thoroughly investigated as possible contributors to the intention and decision to leave general practice early.

1) *Job satisfaction.*

Sibbald et al. [26] found that in 2001 22% of UK GPs intended to leave the profession before reaching the pensionable age; in 1998 this was only 14%. They also found that the overall job satisfaction decreased between those periods and thus they identified overall job satisfaction as a key predictor of GPs’ intention to leave. Other studies demonstrated the same relationship among general practitioners [26,35,52-56].

Job satisfaction scale in survey (α = .73)

The participants responded to eight statements about job satisfaction, originally derived from several studies [57,58]. Likert scales were used, ranging from 1 = completely agree to 5 = completely disagree. An example of the statements is: “I was satisfied with the work I did.”

2) *Emotional exhaustion*

Davidson et al. [33] investigated reasons for the early retirement of Scottish general practitioners. Of the respondents, 36.5% reported pressure of work/exhaustion or burnout as the main reason to retire early. Years later, these were again the most cited reasons [59]. In the Netherlands, the process of burnout among GPs was studied and this revealed that demanding patient contact produces a lack of reciprocity in the GP-patient relationship [60]. The imbalance in relationship experienced by GPs caused feelings of exhaustion over time and initiated the process of burnout [61,62].

Emotional exhaustion scale in survey (α = .92)

In the survey, part of the UBOS [63], a Dutch version of the Maslach Burnout Inventory, was used to measure levels of emotional exhaustion. This scale consisted of seven items, with an answering format ranging from 1 = never to 7 = always. An example of a statement is: “At the end of a working day, I felt empty.”

3) *Subjective workload*

High workload is the principal source of job-related discontent among British doctors, including general practitioners [21,64]. Additionally, a survey among general practitioners in Scotland indicated that 71% of older general practitioners (>55 years) plan to retire at or before the age of 60, with excessive workload cited as the main reason [65].

Subjective workload scale in survey (α = .78)

The participants completed six questions about subjective workload, originally derived from Karasek and Theorell [66]. The answering formats of the items ranged from 1 = never to 4 = always. An example question is: “Did you have to work very fast?”

#### Objective workload/hours worked

Longer reported working hours were associated with lower levels of satisfaction [21,64]. And part time work is associated with generally lower levels of stress and higher levels of job satisfaction than full time working [36,67,68].

The GP survey included two questions about the number of working hours of GPs: ”What was the average of total weekly working hours?” and “How many out-of-hours shifts a year did you have?”

#### External factors

Several external factors may have contributed to the decision to retire.

1) *External control*

There is evidence that physicians are experiencing an increased workload due to external factors, including financial deficits, audits, regulation, administrative policies and procedures. Spickard et al. [69] found that it is important for GPs to have a sense of control over the practice environment and external factors like those mentioned above. In the Netherlands, guidelines have been developed by the Dutch College of General Practitioners (*NHG*) [70].

External control scale in survey (α = .83)

The GP survey included a scale (six statements) about the extent to which GPs experienced a burden caused by external control in six activities, for example, ‘an increasing amount of regulations’. The scale consisted of six items. The items ranged from 1 = no burden at all to 5 = a great burden.

2) *Demands from the government and health insurers*

Government and health insurers create demanding procedures in GPs’ work. For example, in 2006 the Dutch health insurance system changed from a system with privately and publicly insured patients to a system with a basic insurance for all citizens. At the same time, the remuneration system for GPs was changed to a system with a basic capitation fee, differentiated by age and deprivation area and supplemented by a fee-for-service system for each consultation [16,71].

Demands from the government and health insurers scale in survey (α = .81)

The survey contained a scale about the extent to which GPs experienced a burden caused by demands from the government and health insurers, for example ‘their influence on prescription behaviour or numbers of patients’. This scale consisted of four items, of which the answering format ranged from 1 = no burden at all to 5 = a great burden.

3) *Demands from patients, media and society*

The sources of job stress that could lead to early retirement among GPs is also related to increased and inappropriate demands from patients, society and the media [68,69]. There are several studies that suggest that the increasing demands from patients may influence the GP-patient relationship and have a negative impact on GP job satisfaction and mental health [72]. Furthermore, societal developments, such as the changing social status of GPs and the influence of the media (for example, information on the Internet) may influence patients’ demands.

Societal developments scale in survey (α = .81)

The survey included a scale regarding the extent to which GPs experienced burden caused by societal developments, for example ‘negative media reports’. This scale contained five items.

Demands from patients scale in survey (α = .80)

The survey also contained a question about the extent to which GPs experienced burden caused by demands from patients, for example ‘the increasing independence of patients’. This scale consisted of four items. The items in the scales mentioned above all ranged from 1 = no burden at all to 5 = a great burden.

#### Personal reasons

The GP survey included three scales about personal reasons for the respondents to retire before the age of 65.

1) *Health*

Davidson et al. [33] found that for 19.6% of GPs maintaining good health or the desire to retire while still healthy are possible reasons for retirement. Although few studies have investigated the relationship between health and the intention or decision to retire, it is plausible that GPs retire early for health reasons, either due to poor health or because of the prospect of enjoying good health for several years.

Health scale in survey (α = .74)

Four statements highlight which reasons regarding personal health were part of the respondents’ decision to leave general practice. ‘The inability to work’ or ‘the possibility of enjoying several years in good health’ are examples of such reasons. The answers in this scale ranged from 1 = was not part of the decision to 3 = was definitely part of the decision.

2) *Family reasons and time for leisure*

Davidson et al. [33] found that family reasons and time for leisure were reported as the second-most important reason to retire early (26.1%). In a later study, family reasons and the desire for more leisure time were again important factors for considering early retirement [55], which indicates that this reason remained important over time.

Family reasons and time for leisure scale in survey (α = .76)

Five questions in this scale ask which reasons related to life outside the work context (leisure time, family reasons) were part of the respondents’ decision to leave general practice, for example ‘more time for self’ or ‘more time for family’. The answers in this scale ranged from 1 = was not part of the decision to 3 = was definitely part of the decision.

3) *Change of career*

The intention of career change as a reason for leaving general practice early was only mentioned by a small proportion (6.8%) of GPs in Davidson et al. [33]. This implies that career change is not a major reason for GPs to retire early, which is confirmed by Brett et al. [59].

Change of career scale in survey (α = .70)

The participants completed six questions regarding the possibility that career change was part of the decision to leave general practice, for example ‘the desire for a management position or a position outside the medical world’. The answers in this scale ranged from 1 = was not part of the decision to 3 = was definitely part of the decision.

### Analyses

Analyses were made using Stata 11 software. First, descriptive statistics were calculated as well as the mean scores of the scales explained in the preceding paragraphs. To answer the first research question, two-sample t-tests were conducted to test the differences between the mean scores of the different groups (period and gender) on work perception. To answer the second research question, two-sample t-tests were conducted to test the differences between different groups (gender and period) regarding objective workload. To answer the third research question, the mean scores of the scales that measured the external factors and personal reasons were used and two-sample t-tests were conducted to test the differences between the mean scores of the different groups (period and gender). Lastly, to answer the fourth question, the results of research questions one and two were combined with information about the probability of leaving within one year and the retirement age, from a study conducted by Van der Velden & Batenburg [22].

We did not test the relationships and interactions between the work perception, objective workload, external and personal factors and the retirement age or probability to leave, because these relationships have been studied extensively in earlier research. In addition, the number of respondents was too low to conduct regression analysis, especially when disaggregated for gender.

## Results

### Work perception

Table 2 presents the mean scores of the scales that define three constructs of work perception in this survey: job satisfaction, subjective workload and emotional exhaustion. The respondents reported on job satisfaction in both periods. GPs who retired between 1998 and 2002 reported a significantly lower job satisfaction than GPs who retired between 2003 and 2007 (p = 0.000); this difference applies to both male and female GPs. In the first period, male GPs had a significant higher job satisfaction than female GPs (p = 0.004). In addition, male respondents reported significantly less emotional exhaustion in the second period than in the first period (p = 0.000). The difference for female GPs was not significant. There were also differences between male and female GPs in both periods: the emotional exhaustion of male GPs was significantly lower (p = 0.027 and p = 0.045). There were no significant differences regarding subjective workload.

**Table 2:** Mean scores (95 % CI) of three dimensions of work perception of GPs

	**1998-2002**			**2003-2007**			Δ
	**Male**	**Female**	**Total**	**Male**	**Female**	**Total**	
	**(n = 256)**	**(n = 26)**	**(n = 282)**	**(n = 175)**	**(n = 16)**	**(n = 191)**	
***Work perception***							
Job satisfaction (1–6)	1.24^12^	1.04^12^	1.22^1^	1.75^1^	1.55^1^	1.74^1^	+
	(1.20-1.28)	(0.89-1.20)	(1.18-1.26)	(1.67-1.83)	(1.29-1.83)	(1.66-1.81)	
Subjective workload (1–4)	0.75	0.88	0.75	0.73	0.66	0.73	0
	(0.69-0.80)	(0.67-1.09)	(0.71-081)	(0.65-0.81)	(0.42-0.90)	(0.65-0.80)	
Emotional exhaustion (1–7)	3.05^12^	3.52^2^	3.09^1^	2.24^12^	2.87^2^	2.30^1^	-
	(2.92-3.18)	(3.07-3.98)	(2.97-3.22)	(2.06-2.42)	(2.12-3.63)	(2.12-2.47)	

^1^ difference between periods (p < .05) (*t*-test).

^2^ difference between men and women (p < .05) (*t*-test).

### External factors and personal reasons for leaving general practice

There are different factors that may influence a GP’s decision to leave general practice. Table 3 presents the mean scores of external factors burdening GPs and personal reasons for retirement. Male GPs experienced the highest burden from external control in both periods (1998–2002 and 2003–2007), while female GPs experienced the highest burden from demands by patients in the first period and from external control in the second period. For both male and female GPs, in the second period societal developments became less important than demands from the government and health insurers. Additionally, family reasons/wanting time for leisure was the most important personal reason for both male and female GPs in both periods. Health was rated as the second most important reason and career change as the third. However, for female GPs in the second period, health was cited as a less important reason for leaving general practice than career change.

**Table 3:** Mean scores (95 % CI) of factors influencing the decision to retire

	**1998-2002**			**2003-2007**			Δ
	**Male**	**Female**	**Total**	**Male**	**Female**	**Total**	
	**(n = 256)**	**(n = 26)**	**(n = 282)**	**(n = 175)**	**(n = 16)**	**(n = 191)**	
***External factors***							
External control (1–5)	2.63	2.75	2.64	2.62	2.51	2.61	0
	(2.56-2.69)	(2.53-2.97)	(2.58-2.70)	(2.50-2.73)	(2.02-3.00)	(2.50-2.72)	
Demands from government and health insurers (1–5)	2.35^1^	2.44	2.35^1^	2.11^1^	2.23	2.12^1^	-
	(2.29-2.40)	(2.24-2.64)	(2.30-2.41)	(1.98-2.24)	(1.76-2.71)	(2.00-2.24)	
Societal developments (1–5)	2.54^1^	2.59^1^	2.55^1^	2.07^1^	1.99^1^	2.06^1^	-
	(2.48-2.60)	(2.37-2.82)	(2.49-2.61)	(1.97-2.17)	(1.65-2.33)	(1.97-2.16)	
Demands from patients (1–5)	2.56^1^	2.76^1^	2.58^1^	2.26^1^	2.28^1^	2.26^1^	-
	(2.49-2.62)	(2.52-3.00)	(2.52-2.64)	(2.16-2.35)	(1.95-2.61)	(2.17-2.35)	
***Personal reasons***							
Career change (1–3)	1.26^1^	1.31	1.27^1^	1.15^12^	1.38^2^	1.17^1^	-
	(1.21-1.32)	(1.12-1.50)	(1.22-1.32)	(1.10-1.20)	(1.01-1.76)	(1.12-1.23)	
Health (1–3)	1.57^1^	1.77^1^	1.59^1^	1.31^1^	1.28^1^	1.30^1^	-
	(1.50-1.64)	(1.48-2.06)	(1.52-1.66)	(1.23-1.39)	(1.04-1.52)	(1.23-1.38)	
Family reasons and time for leisure (1–3)	1.87^1^	1.92	1.87^1^	1.74^1^	1.57	1.72^1^	-
	(1.79-1.94)	(1.67-2.17)	(1.80-1.95)	(1.66-1.82)	(1.28-1.87)	(1.64-1.81)	

^1^ difference between periods (p < .05) (*t*-test).

^2^ difference between men and women (p < .05) (*t*-test).

The mean score of several external factors changed from one period to the next. Male GPs experienced less demand from government and health insurers in the second period than in the first period (p = 0.000). Both men (p = 0.000) and women (p = 0.002) experienced significantly less influence of societal developments in the second period compared to the first. Furthermore, both men (p = 0.000) and women (p = 0.017) experienced less burden of demands from patients in the second period.

In addition, the mean scores of several personal reasons influencing the decision to leave general practice changed from one period to the next. For male GPs, change of career was less important as a reason to retire in the second period than in the first period (p = 0.004). For female GPs, there was no difference between the two periods regarding career change as a reason for leaving general practice, but there was a difference between male and female GPs in the second period regarding career change (p = 0.018): female GPs reported career change as being more important than male GPs. Both male GPs (p = 0.000) and female GPs (p = 0.022) reported health as a more important reason for leaving general practice in the first period than in the second period. The mean scores of the family reasons and wanting time for leisure as a personal reason for retiring show that these factors were less important for male GPs in the second period than in the first period (p = 0.028). There was also a difference for female GPs, but this was not significant.

### Relating work perception, reasons for retirement and the probability of leaving

The probability of leaving within one year decreased from one period to the next for both male and female GPs, with a stronger decrease for female GPs. Furthermore, the retirement age of Dutch GPs increased in the second period. There were not only fewer GPs leaving in the second period, but they were also older when they left. It is important to establish what factors are at play in these changes.

#### Female GPs

The work perception of female GPs changed between 1998–2002 and 2003–2007. Their job satisfaction was higher in the second period and their degree of emotional exhaustion was somewhat lower in the second period (but not significantly so). However, during both periods their degree of emotional exhaustion was higher than that of male GPs. There was no change in the experienced subjective workload.

For female GPs, external factors that were burdening GPs and may have contributed to the decision to retire were less important in the second period than in the first period. The external factors were external control (not significant), demands from the government and health insurers (not significant), and demands from society (significant) and patients (significant).

Moreover, personal reasons that may have contributed to the decision to retire were somewhat less important for female GPs in the second period. Personal health as a reason to retire early decreased from one period to the next. In both periods career change, family reasons and time for leisure were reasons to retire from general practice; however, they did not become more or less important in the second period.

#### Male GPs

There were significant changes in the work perception of male GPs between 1998–2002 and 2003–2007. Their job satisfaction was higher and their degree of emotional exhaustion was lower in the second period. However, their subjective workload did not change.

For male GPs, two external factors that were burdening GPs and that may have contributed to the decision to retire were significantly less important in the second period, namely demands from the government and demands from health insurers. In addition, demands from patients and developments in society became less important in the second period.

Moreover, in the second period personal reasons that may have contributed to the decision to retire were less important for male GPs. Personal health as a reason to retire decreased from one period to the next. The same is true for career change, for which there was also a difference between male and female GPs in the second period, in which career change proved less important for male GPs. For male GPs, family and time for leisure as reasons to retire were also lower in the second period.

### Objective workload and probability of leaving

For both male and female GPs, there was no decrease in subjective workload from 1998–2002 to 2003–2007, but there was a higher job satisfaction and less emotional exhaustion in the second period. In Table 4, the objective workload is depicted for male and female GPs in both periods. The objective workload decreased significantly for male GPs, as they had significantly fewer out-of-hours shifts in the period 2003–2007 than in the period 1998–2002.

**Table 4:** Objective workload of self-employed GPs, mean (95 % CI)

	**1998-2002**			**2003-2007**			Δ
	**Male**	**Female**	**Total**	**Male**	**Female**	**Total**	
	**(n = 256)**	**(n = 26)**	**(n = 282)**	**(n = 175)**	**(n = 16)**	**(n = 191)**		
***Objective workload***							
Number of working hours	52.0^2^	45.6^2^	51.4	51.3^2^	39.8^2^	50.6	0
	(50.4-53.6)	(39.1-52.2)	(49.8-53.0)	(49.1-53.5)	(27.4-52.2)	(48.3-52.8)	
Number of out-of-hours shifts (evening) per year	51.3	36.6	50.2^1^	33.0^1^	29.5	32.9^1^	-
	(47.2-55.4)	(27.2-45.9)	(46.3-54.1)	(26.0-40.0)	(−9.3-68.3)	(26.1-39.6)	
Number of out-of-hours shifts (weekend) per year	11.7^1^	9.8	11.8^1^	9.8^1^	13.2	10.0^1^	-
	(11.2-12.7)	(7.8-11.7)	(11.0-12.5)	(8.6-11.0)	(−3.9-30.2)	(8.7-11.2)	

^1^ difference between periods (p < .05) (*t*-test).

^2^ difference between men and women (p < .05) (*t*-test).

Furthermore, there were no reasons for leaving general practice that were *more* important in the second period; by contrast, the majority of the external factors and personal reasons became *less* important. However, for female GPs the objective workload did not provide any evidence for the stronger decrease in the probability of leaving.

## Discussion

This study investigated work perception, objective workload, external factors burdening GPs and personal reasons for leaving general practice among two groups of retired general practitioners: those that left general practice in 1998–2002 and those that left in 2003–2007. This was investigated to account for both the difference between these two periods in the probability for leaving general practice within one year, especially for female GPs, and the different retirement ages in both periods.

The work perception of both male and female GPs was different for the period 1998–2002 and the period 2003–2007: both groups experienced a higher job satisfaction and a lower degree of emotional exhaustion, although the difference for female GPs was not significant. There was no difference in subjective workload, but the objective workload was lower in the second period, especially for male GPs because they had a lower number of out-of-hours shifts (evening and weekend) per year.

Furthermore, external factors that were experienced as a burden and that may have contributed to the decision to retire were less important for GPs in the second period than in the first period, with more significant differences for male GPs. In addition, personal reasons that could contribute to the decision to retire were somewhat less important for female and male GPs in the second period.

The probability of leaving within one year was lower in the period 2003–2007 than in the period 1998–2002 for both male and female GPs; however, this decrease was stronger for female GPs. Nevertheless, in the second period female GPs still had a higher probability of leaving general practice within a year than male GPs. Additionally, the retirement age of both male and female GPs was higher in the second period (M: 58.8, F: 49.1) than in the first period (M: 56.5, F: 47.0). Not only were there fewer GPs who retired from general practice in the period 2003–2007 than in the period 1998–2002, but the retirement age was also higher in 2003–2007. This reflects the changing wider societal trend of the increasing will to work until 65 (statutory retirement age) [15]. To evaluate the policies that have been implemented to encourage later retirement, it is useful to understand the link between the factors influencing the decision to retire and the actual turnover in the medical and GP workforce. Although the subjective workload for both male and female GPs was not lower in the second period, the objective workload decreased from one period to the next for male GPs. In addition, both male and female GPs had a higher job satisfaction and less emotional exhaustion in the second period. Furthermore, a majority of external factors and personal reasons that may have contributed to the decision to retire were less important contributors to this decision in the second period. The general probability of leaving has decreased, but we found no evidence why this decrease was stronger for female GPs than for male GPs. However, these findings underscore the differences between male and female GPs. To conduct workforce planning accurately and respond to the existing gender differences, it is important to understand the different reasons male and female GPs have to retire. Therefore, there is need for gender disaggregated data collection. The information in the NIVEL GP database is disaggregated by gender.

There are differences between the two periods 1998–2002 and 2003–2007 regarding the moment and probability of retirement, as well as the reasons influencing the decision to retire. The introduction of several workload reduction measures in the Netherlands at the start of the 21st century, have possibly contributed to these differences. These measures were implemented to reduce high workload among GPs: the nationwide introduction of central GP services for evening, night and weekend service and the introduction of general practice nurses in GP practices. These measures were discussed more extensively in the background section of this article [41,42].

According to earlier research, the number of evening, night and weekend shifts per GP and the experienced workload reduced significantly after the introduction of large-scale central GP services [44,48]. Also the findings of the present study show that there was a decrease in evening, night and weekend shifts (Table 4).

A possible reduction in GP workload was expected because of the introduction of the general practice nurse if GPs could focus on their core tasks and the provision of care was shared with other health professionals. During the present study, we acquired additional information about these practice nurses. We found that in the period 1998–2002, 6.7% of the respondents mentioned that a practice nurse was working in their practice, while this percentage had risen to 37.6% in 2003–2007.

It was confirmed by the results of the present study that these two workload reduction measures (central GP services for out-of-hours care and practice nurses) were more common in the second period.

Another possible explanation for the differences found between both periods are changing financial circumstances for GPs. Self-employed GPs can only retire from general practice when it is also financially attractive for them. The respondents in this study answered two questions about remuneration that could have influenced their decision to leave general practice: *‘There were no financial stimulants to stay’* and ‘*It was not necessary to continue working for financial reasons.’* However, the answers to these questions were contradictory and therefore no conclusions could be drawn about the influence of remuneration. Landon et al. [73] did found that the income of physicians is not related to the decision of retirement.

### Strengths and limitations of the study

This study has a number of limitations. The response rates of the surveys are moderate (60 and 54%). Analysis of the differences in personal characteristics between respondents and the population of retired GPs in these periods shows that the respondents were somewhat older than the overall population of GPs that left general practice. While the sample remained reasonably representative in terms of demographic characteristics, it cannot be ruled out that it was the most dissatisfied group that responded. However, it seems unlikely that the survey results do not represent the group of retired GPs. For the probability of leaving within one year and the retirement age, data from the entire GP population were used, and generally these data were comparable to the results of our survey.

A second limitation is that GPs who left general practice before they reached retirement age were asked retrospectively to mention their reasons for leaving general practice. It is possible that their answers were influenced by the time elapsed. However, we expect this bias to be minimal, because it has been shown that reports on jobs that respondents had 5 years ago do not exhibit greater unreliability than reports on jobs of a few months ago [50,51]. Our respondents received the survey a maximum of five years after they retired.

After cleaning up the data, there were relatively few female GPs left, especially in the second survey. For this reason, the 95% confidence intervals for female GPs were mostly quite large. This was due to the fact that there are fewer self-employed female GPs than self-employed male GPs in the Netherlands (an average of 21.4% female self-employed GPs in 1998–2002 and an average of 27.1% in 2003–2007). Despite the small numbers differences were found, and it is possible that we would have found more and larger differences if we had had more female respondents.

A strength of this study is the use of results from the two periods, because these were the periods approximately before and after two major workload reduction measures were introduced in the Netherlands. Another strength of this study is the use of actual turnover as an outcome measure, rather than the intention to leave general practice. Other research suggests that intentions to retire do not necessarily translate into action [26,74]. In several studies the intention to retire represents the same construct as job satisfaction. According to Rittenhouse [40], job satisfaction is a better predictor of the intention to leave general practice than of actual turnover. In our surveys, both actual turnover and job satisfaction were used.

### Implications for policy and future research

The reduction of the high (subjective ánd objective) workload of GPs is an important goal, because a high workload may lead to a high probability of early retirement and of leaving general practice within one year. Both may have consequences for patients, and it is likely that the quality of care will suffer as a result [75]. At the very least, it is important information for planning the GP workforce to meet the future demand for care. After all, projections of the future outflow of GPs are based on the retirement age of GPs in the past. Moreover, due to the high cost of training and the relatively long training period for physicians, it may be beneficial to stimulate physicians to retire at a later age. Several studies have shown that the proportion of physicians working beyond the age of 60 has fallen in most European countries over the past decade [25-27]. Until recently, few OECD countries had implemented or planned specific policies to address this issue [28]. A greater understanding of the link between the factors influencing the decision to retire and actual turnover would therefore be useful in the development of policies to encourage later retirement. The results of this study indicate that the implementation of workload reduction policies, such as reducing out-of-hours shifts, are an important contributor to encouraging policies for GPs to retire later.

There is evidence that physicians are experiencing an increased workload due to external factors, including financial deficits, audits, regulation, administrative policies and procedures [36,69]. According to the results of this study, male GPs experienced the highest burden from external control in both periods (1998–2002 and 2003–2007) and female GPs experienced the highest burden from external control in the second period. These results show that external control (as a potential contributor to the decision to retire) did not (significantly) decrease between two periods. Implementing policy that decreases external control, by for example decreasing the administrative burden for GPs, could potentially reduce GPs’ workload even more.

Since 2006, a new health insurance act has been in force in the Netherlands. Our study only covers GPs that left general practice during two periods: 1998–2002 and 2003–2007. We expect that the introduction of the new health insurance act did not contribute to the decision to leave between 2003 and 2007. However, the first reactions by GPs to these system changes have not been very positive. GPs expect the new health insurance act to lead to increased administration and regulations, which will cause an increase in non-patient related activities [76,77] Therefore, it would be interesting to use the same survey for GPs that left general practice during the next five year period: 2008–2012. It would be especially interesting to investigate whether the administrative burden has changed and whether this has had any effect on the work perception, the reasons for leaving general practice and the probability of leaving within one year.

## Conclusion

The results of this study suggest that the decrease in the probability of leaving general practice within one year and the increase in the retirement age have been caused by not only a decrease in the objective workload and a change in the work perceptions of GPs, but also by external factors and personal factors. It was confirmed by the results of the present study that the two workload reduction measures introduced by the Dutch government (central GP services for out-of-hours care and practice nurses) were more common in the second period. These changes therefore likely have been influenced by the implementation of these policies.

As stated in the introduction of this article, countries have a variety of additional policy instruments at their disposal to influence the supply of physicians. Based on the results of this study, we consider workload reduction policies are the most useful instruments to control retention and retirement.

## Abbreviations

GP: General practitioner; FTE: Full-time equivalent.

## Competing interests

The authors declare that they have no competing interests.

## Authors' contributions

LVDV and PH composed the questionnaire and collected the data for the first research period. MVG revised the questionnaire and collected the data for the second research period. MVG also conducted the comparative analyses and drafted and revised the manuscript. PH helped to interpret the analyses and helped draft and revise the manuscript. All authors read and approved the final manuscript.

## Pre-publication history

The pre-publication history for this paper can be accessed here:

http://www.biomedcentral.com/1472-6963/12/467/prepub

## Supplementary Material

Additional file 1 Probability of leaving general practice within one year, by age, gender and period. Click here for file
