# Supplementary material for: Motives for early retirement of self-employed GPs in the Netherlands: a comparison of two time periods
Source: BMC Health Serv Res. 2012 Dec 18;12:467. doi: 10.1186/1472-6963-12-467 (PMC3541202; doi:10.1186/1472-6963-12-467)
Supplement: Additional file 1 — Probability of leaving general practice within one year, by age, gender and period. [file 1472-6963-12-467-S1.pdf]

Additional file 1. Probability of leaving general practice within one year, by age, gender and period.

	1998-2002				2003-2007				Δ			
Age	<=54	55-59	60-64	Total	<=54	55-59	60-64	Total	<=54	55-59	60-64	Total
Male	1.5%	7.4%	35.2%	3.3%	0.7%	5.2%	21.3%	3.0%	-0.8%	-2.2%	-13.9%	-0.3%
Female	2.3%	11.4%	34.8%	2.7%	1.3%	5.3%	21.3%	1.7%	-1.0%	-6.1%	-13.4%	-1.0%
Total	1.7%	7.6%	35.1%	3.2%	0.9%	5.2%	21.3%	2.7%	-0.8%	-2.4%	-13.9%	-0.5%

Source: Van der Velden LFJ, Batenburg RS: *Stoppen als huisarts: trends in aantallen en percentages. Een analyse van de huisartsenregistratie over de periode 1998-2007.[Leaving general practice: Trends in numbers and percentages. An analysis of the GP registration for the period 1998-2007.]*. Utrecht: NIVEL; 2009.
